# Frequency Specific Effects of* ApoE ε*4 Allele on Resting-State Networks in Nondemented Elders

**DOI:** 10.1155/2017/9823501

**Published:** 2017-03-15

**Authors:** Ying Liang, Zhenzhen Li, Jing Wei, Chunlin Li, Xu Zhang, Alzheimer's Disease Neuroimaging Initiative

**Affiliations:** ^1^School of Biomedical Engineering, Capital Medical University, Beijing 100069, China; ^2^Beijing Key Laboratory of Fundamental Research on Biomechanics in Clinical Application, Capital Medical University, Beijing 100069, China

## Abstract

We applied resting-state functional magnetic resonance imaging (fMRI) to examine the Apolipoprotein E (*ApoE*) *ε*4 allele effects on functional connectivity of the default mode network (DMN) and the salience network (SN). Considering the frequency specific effects of functional connectivity, we decomposed the brain network time courses into two bands: 0.01–0.027 Hz and 0.027–0.08 Hz. All scans were acquired by the Alzheimer's Disease Neuroscience Initiative (ADNI). Thirty-two nondemented subjects were divided into two groups based on the presence (*n* = 16) or absence (*n* = 16) of the* ApoE ε*4 allele. We explored the frequency specific effects of* ApoE ε*4 allele on the default mode network (DMN) and the salience network (SN) functional connectivity. Compared to *ε*4 noncarriers, the DMN functional connectivity of *ε*4 carriers was significantly decreased while the SN functional connectivity of *ε*4 carriers was significantly increased. Many functional connectivities showed significant differences at the lower frequency band of 0.01–0.027 Hz or the higher frequency band of 0.027–0.08 Hz instead of the typical range of 0.01–0.08 Hz. The results indicated a frequency dependent effect of resting-state signals when investigating RSNs functional connectivity.

## 1. Introduction

Apolipoprotein E (*ApoE*) *ε*4 allele has been proved to be a risk gene of late-onset Alzheimer's Disease (AD) [1]. It may cause a variety of functional and structural changes in human brain [2, 3] and is associated with greater amyloid-*β* (A*β*) accumulation and neurofibrillary tangles than *ε*3 [4]. It already shows a subtle decline in episodic memory many years before the development of dementia [5]. As there is no effective treatment of AD now, looking for sensitive and reliable biomarkers in earliest stages is very important.

Imaging technologies like functional magnetic resonance imaging (fMRI) offered an opportunity to detect the effects of gene on the brain function by blood oxygenation level dependent (BOLD). Nowadays, emerging computational tools made it possible to study brain networks instead of single brain region in a stereo vision. Specific cortical regions which are spatially separated from brain functional networks to complete cognitive task [6] and neurodegenerative diseases such as AD target specific large-scale brain networks [7]. It has been suggested that two resting-state networks (RSNs), salience network (SN) and the default mode network (DMN) [8], where the atrophy caused by dementia is largely concentrated, play an essential role in AD. A few previous studies have suggested ApoE *ε*4 allele may affect the activity of DMN and SN [9–11].

Previous studies mainly examined resting-state fMRI activities in the frequency between 0 and 0.08 Hz as this frequency band was thought to be associated with neuronal fluctuations [12], but more and more researchers suggested that functional connectivity may be frequency specific [13]. Complex activities in human brain produce different neuronal firing rate; different frequency may correspond to different cognitive courses [14]. A fMRI combined electroencephalography (EEG) study suggested that each resting-state network corresponds to a specific frequency rhythm [15]. Although abnormal resting-state networks were observed in both normal aging and* ApoE ε*4 carriers [9, 16–18], whether the abnormalities are frequency specific is still unknown. Han et al. used 0.027 Hz as dividing level of the low-frequency fluctuations (ALFF) and found that the ALFF abnormalities showed disparate spatial patterns in each frequency band [19]. Many studies also have reported the fMRI signals at both lower and higher frequencies contain important physiological significance; they may lose information if they are considered as a whole [20, 21].

In the present study, we aimed to utilize resting-state fMRI to examine the* ApoE ε*4 allele effects on functional connectivity of DMN and SN. Considering the frequency specific effects of functional connectivity, we decomposed the brain network time courses into two bands: 0.01–0.027 Hz and 0.027–0.08 Hz. We sought to determine (1) whether the* ApoE ε*4 carriers show abnormal resting-state network functional connectivity; (2) whether the functional connectivity abnormalities are frequency specific.

## 2. Materials and Methods

### 2.1. Subjects

All data used in this current study were obtained from the ADNI database (http://adni.loni.usc.edu). For the current study we randomly included a total of 16* ApoE ε*4 carriers (genotypes ɛ4/ɛ4 and ɛ4/ɛ3) and 16 age- and gender-matched noncarriers (genotype ɛ3/ɛ3) from ADNI. Individuals with the ɛ2 allele were excluded due to its possible protective effects [22]. Only the baseline 3T scans of each subject was utilized. Inclusion criteria of all subjects in this study were aged between 55 and 80, with a Mini Mental State Examination (MMSE) score ≥ 24, lack of MCI or dementia, and a Clinical Dementia Rating-Sum of Boxes (CDR-SB) score of 0. The study was approved by the Institutional Review Boards of all of the participating institutions of ADNI, and informed written consent was written from all participants.

### 2.2. Neuropsychological Tests and* ApoE* Genotyping

The cognitive function scores used in this study were downloaded from the ADNI database. In this study, we focused on the results of general cognitive ability tests using Mini Mental State Examination (MMSE) and episodic memory using Ray Auditory Verbal Learning Test (RAVLT) (Table 1).* ApoE* genotyping was analysed from DNA samples of each participant's blood cells, applying an* ApoE* genotyping kit.

### 2.3. Data Acquisition

Subjects were scanned using a 3.0-Tesla Philips MRI scanner. Resting-state fMRI images were obtained using an echo-planar imaging (EPI) sequence (repetition time (TR) = 3000 ms, echo time (TE) = 30 ms, flip angle = 80°, number of slices = 48, voxel size = 3 mm × 3 mm × 3 mm, slice thickness = 3.3 mm, and voxel matrix = 64 × 64).

### 2.4. Imaging Preprocessing

Image preprocessing and analysis were performed using Statistical Parametric Mapping (SPM8, http://www.fil.ion.ucl.ac.uk/spm), Resting-State fMRI Data Analysis Toolkit (REST; Song et al., http://restfmri.net), and the Data Processing Assistant for resting-state fMRI (DPARSF, Yan and Zang; http://restfmri.net/forum/DPARSF). The first 10 volumes of the rest scans of each subject were removed for the signal equilibrium and for subject's adaptation to the scanning noise. The functional images left were preprocessed including slice timing, motion correction (exclusion threshold was set as 3 mm for the linear translation), spatial normalization to template in Montreal Neurological Institute (MNI) coordinate space, and resampling with 3 × 3 × 3 mm^3^. Then all images were smoothed with a 4 mm full-width half-maximum (FWHM) Gaussian kernel. Furthermore, the resting-state fMRI data were linearly detrended and processed with regression correction for several nuisance covariates including six motion parameters, white matter signal, global mean signal, and cerebrospinal fluid signal. After preprocessing, data of 2 subjects (1* ApoE ε*4 carrier; 1* ApoE ε*4 noncarrier) were excluded from the following analyses due to excessive motion.

### 2.5. Frequency Division and Functional Connectivity Calculation

After preprocessing, we used low-pass/band-pass filters in REST to generate different data sets including three specific frequency bands: 0.01–0.027 Hz, 0.027–0.08 Hz, and the typical range of 0.01–0.08 Hz. We followed the literature [23] to define seed regions of interest (ROI) of the DMN and SN; 15 ROIs were derived as a 6 mm sphere around the coordinates in MNI space (Table 2). Within the DMN, nine regions were investigated: medial prefrontal cortex (mPFC), left lateral parietal (lLP), right lateral parietal (rLP), posterior cingulate cortex (PCC), left inferior temporal (liTmp), right inferior temporal (riTmp), medial thalamus (mdThal), left posterior cerebellum (lpCBLM), and right posterior cerebellum (rpCBLM). In the SN, six regions were investigated: left anterior cingulate cortex (lPG-ACC), right anterior cingulate cortex (rPG-ACC), right ventral anterior cingulate cortex (rSG-ACC), putamen (Put), left insula (lIns), and right insula (rIns). Then functional connectivity matrix of each network was produced by averaging the Blood-Oxygen-Level-Dependent (BOLD) signal across all voxels within these ROIs and computing Pearson's correlation coefficients. The correlation coefficients were then transformed into *z* scores using Fisher *r*-to-*z* transformation.

### 2.6. Statistical Methods

Data were analysed using statistical software (SPSS, version 22.0). Two-sample* t*-tests were used to examine the significance of group differences in age, education, and neuropsychological scores, as well as the group differences of RSNs functional connectivity matrix in different frequency bands (0.01–0.027 Hz, 0.027–0.08 Hz, and 0.01–0.08 Hz). Gender data were calculated using *χ*^2^-test. Pearson's correlation analyses were performed between the RSNs functional connectivity and the neuropsychological scores to explore whether the functional connectivity in different frequency bands is associated with the cognitive function.

## 3. Results

### 3.1. Demographic and Neuropsychological Measurements

The demographic characteristics and neuropsychological test scores of the ɛ4 carrier and noncarrier groups are shown in Table 1. There is no difference in age, gender, or education between the two groups of carriers and noncarriers. Scores on the MMSE and RAVLT were found to be significantly decreased in* ApoE ε*4 group compared with noncarrier group (Table 1).

### 3.2. Functional Connectivity

**Figure 1:**
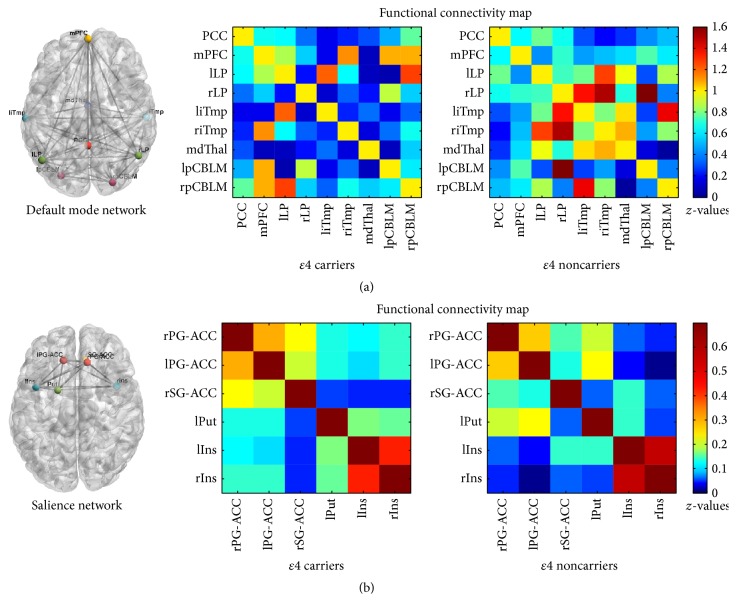
The default mode network and salience network nodes and functional connectivity map (0.01–0.08 Hz) of the *ε*4 carriers and noncarriers. Correlation matrix of all ROI pairs in each network.

Figure 1 shows the RSNs ROIs and the two-sample *t*-test results of the connectivity maps of the two networks in each frequency band, respectively. We found some functional connectivities were sensitive to specific frequency band. In the DMN, the functional connectivity between the PCC and lLP, as well as the liTmp and rpCBLM connectivity, showed no significant group differences in the typical frequency band of 0.01–0.08 Hz but significant differences in the lower frequency band of 0.01–0.027 Hz (Table 3). In the SN, the connectivity between lPG-ACC and rSG-ACC, the connectivity between left insula and right insula, the connectivity between putamen and right insula all showed no significant differences in the typical frequency band of 0.01–0.08 Hz. But the first two connectivity group differences become significant in the lower frequency band of 0.01–0.027 Hz while the latter was significant in the frequency band of 0.027–0.08 Hz (Table 4).

In all the significant group differences, the DMN functional connectivities were decreased in the* ApoE ε*4 carriers compared with noncarriers, while the SN connectivities were increased in the* ApoE ε*4 carriers compared with noncarriers (Figure 1).

### 3.3. Correlation

**Figure 2:**
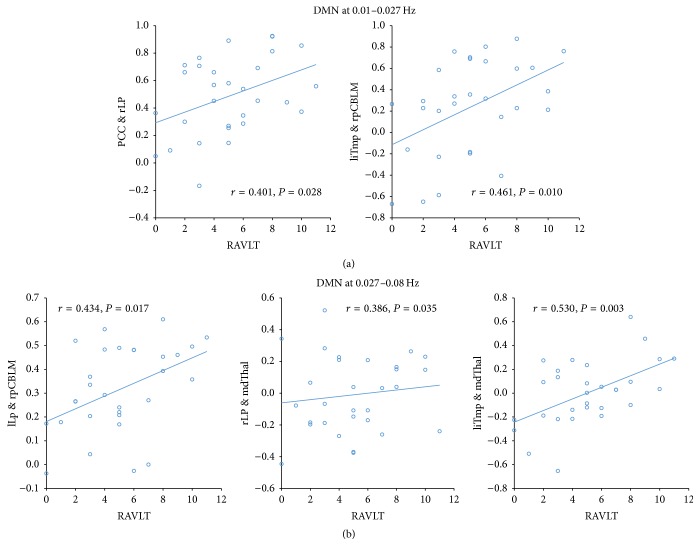
Relationship between altered connectivity and cognition at specific frequency bands in default mode network (0.01–0.027 Hz and 0.027–0.08 Hz).

In the DMN, at the frequency band of 0.01–0.027 Hz, we found significant positive correlations between the PCC and rLP connectivity (*r* = 0.40, *P* = 0.028) and liTmp-rp and rpCBLM connectivity (*r* = 0.46, *P* = 0.010) with RAVLT scores. At the frequency band of 0.027–0.08 Hz, the rLP and rpCBLM connectivity (*r* = 0.43, *P* = 0.017), the liTmp-rp and mdThal connectivity (*r* = 0.39, *P* = 0.035), and rLP and mdThal connectivity (*r* = 0.53, *P* = 0.003) showed significantly positive correlations with the RAVLT scores (Figure 2).

**Figure 3:**
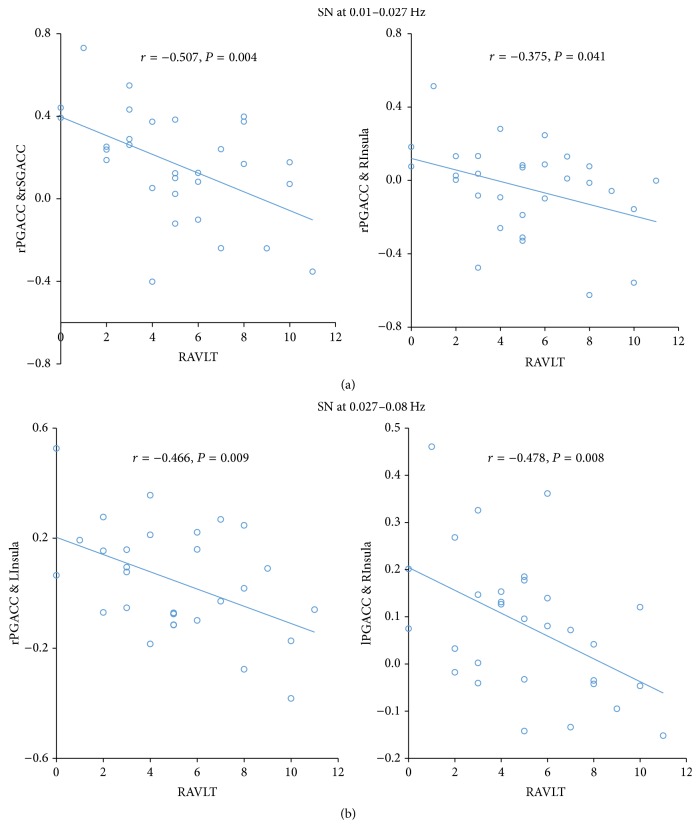
Relationship between altered connectivity and cognition at specific frequency bands in salience network (0.01–0.027 Hz and 0.027–0.08 Hz).

In the SN, at the frequency band of 0.01–0.027 Hz, we found significant negative correlations between the rPG-ACC and rSG-ACC connectivity (*r* = −0.51, *P* = 0.004) and rPG-ACC and rIns connectivity (*r* = −0.38, *P* = 0.041) with RAVLT scores. At the frequency band of 0.027–0.08 Hz, the rPG-ACC and lIns connectivity (*r* = −0.47, *P* = 0.009) and lPG-ACC and rIns connectivity (*r* = −0.48, *P* = 0.008) showed significantly negative correlations with the RAVLT scores (Figure 3).

## 4. Discussion 

In the current study, we examined the functional connectivity changes in DMN and SN in the nondemented* ApoE ε*4 carriers at three different frequency bands (the typical range of 0.01–0.08 Hz, 0.01–0.027, and 0.027–0.08 Hz). Generally, the DMN functional connectivities were decreased while the SN connectivities were increased in the* ApoE ε*4 carriers compared with noncarriers. Importantly, we found that many functional connectivities showed significant differences at the lower frequency band of 0.01–0.027 Hz and the higher frequency band of 0.027–0.08 Hz instead of the typical range of 0.01–0.08 Hz. The results indicated a frequency dependent effect of resting-state signals when investigating RSNs functional connectivity.

Possession of *ε*4 allele disrupts the cognition, especially episodic memory at an early time [24]. We also found a significant decline of the general cognitive ability and episodic memory in *ε*4 carriers, which illustrated that the *ε*4 allele may affect the cognition long before the conversion to AD. More and more studies proved that brain cognitive functions were performed on the base of specific resting-state networks such as DMN and SN [25]. RSNs and their functional connectivity patterns have already shown a potential power of disease diagnosis and prediction [10, 26].

DMN and SN abnormalities were widely found in AD studies, implying that early detection of RSNs changes can offer opportunities to distinguish AD patients from healthy people in early stage [27, 28]. Comparing to the *ε*4 noncarriers, our results showed that several functional connectivities in DMN, such as PCC-lLP connectivity, were significantly reduced in the *ε*4 carriers. Meanwhile, the SN showed widely increased functional connectivity between regions like lPG-ACC and rSG-ACC. These changing trends of functional connectivity we found in the *ε*4 carriers were consistent with the results of early AD [8, 29]. Functional connectivity describes the degree of the dynamic and synchronized oscillations between brain regions. These imaging biomarkers may help a better understanding of early disease pathogenesis and measuring genetic effects on brain, indicated by the significant correlations between the frequency specific functional connectivity and RAVLT scores in our results.

Exploring the high risk genotypes of diseases like AD may reveal the early disease-causing effects. With the development of novel algorithms and theories, nowadays we can correlate the neuroimaging data with the genetic data and discover how genotype affecting brain function and connectivity just as the phonotype, and to identify risks for neurological and psychiatric diseases [30]. The frequency characteristics of functional connectivity may be distinct for different brain networks [13]. The typical range of 0.01–0.08 Hz has its limitations. More and more researches indicate that the fMRI signals contain important physiological significance at specific frequency bands [21, 31, 32]. Moreover, these studies suggested that different frequency bands may play different role in the low-frequency fluctuations [33]. It is important to note that each frequency bands of neuronal oscillations are produced by different oscillators with distinct physiological functions and properties [6].

In the current study, we took advantages of the frequency characteristics of the resting-state fMRI signals to investigate the *ε*4 allele effects on the DMN and SN. We found that some functional connectivities were not sensitive to the typical frequency band of 0.01–0.08 Hz, but when we segmented the frequency band into 0.01–0.027 Hz and 0.027–0.08 Hz group differences emerged. The results indicated that the abnormalities of brain functional connectivity in* ApoE ε*4 carriers are associated with the choice of specific frequency bands. Our result supported that the RSNs functional connectivity can be modulated by frequency band. To the best of our knowledge, this is the first study on the frequency specific effect of the* ApoE ε*4 allele on the RSNs functional connectivity.

There are also some limitations in our study. Although we found a frequency specific effect on the RSNs functional connectivity, the nature of these effects remains unclear. Future studies are necessary to investigate the physiological significance of the frequency specific effects.

## 5. Conclusions

In conclusion, we used genetic neuroimaging methods and found alterations of both DMN and SN functional connectivity in* ApoE ε*4 allele carriers. Our results supported that the RSNs functional connectivity can be modulated by frequency band and emphasized the importance of considering frequency specific effects when investigating the genotypical effect on the brain function.

## Figures and Tables

**Table 1:** Demographic and neuropsychological characteristics of *ApoE ε*4 carriers and noncarriers.

	*ApoE* *ε*4 carriers (*n* = 16)	*ApoE* *ε*4 noncarriers (*n* = 16)	*T*-value (*χ*^2^)	*P*
Age (years)	64.13 ± 6.59	63.13 ± 4.86	0.49	0.629
Education (years)	10.75 ± 3.15	10.63 ± 2.45	0.13	0.901
Gender (M/F)	7/9	8/8	0.12	0.723
MMSE	26.50 ± 2.03	28.75 ± 1.00	−3.97	<0.001^*∗∗∗*^
RAVLT	3.19 ± 2.08	7.00 ± 2.31	−4.98	<0.001^*∗∗∗*^

Values are mean ± standard deviation or numbers of participants. The differences in demographics and neuropsychological scores between the two groups were tested for significance with two-sample *t*-tests. The *P* value for gender distribution in the two groups was obtained using a Chi-square test. ^*∗∗∗*^*P* < 0.01. MMSE, Mini-Mental Status Examination; RAVLT, Ray Auditory Verbal Learning Test.

**Table 2:** Regions and MNI coordinates of the DMN and SN.

ROI	MNI coordinates
*Default mode network*	
Posterior cingulate cortex (PCC)	(0, −51, 29)
Medial prefrontal cortex (mPFC)	(0, 61, 22)
Left lateral parietal (lLP)	(−48, −66, 34)
Right lateral parietal (rLP)	(53, −61, 35)
Left inferior temporal (liTmp)	(−65, −22, −9)
Right inferior temporal (riTmp)	(61, −21, −12)
Medial thalamus (mdThal)	(0, −9, 7)
Left posterior cerebellum (lpCBLM)	(−28, −82, −32)
Right posterior cerebellum (rpCBLM)	(26, −89, −34)
*Salience network*	
Right anterior cingulate cortex (rPG-ACC)	(12, 32, 30)
Left anterior cingulate cortex (lPG-ACC)	(−13, 34, 16)
Right ventral anterior cingulate cortex (rSG-ACC)	(10, 34, −6)
Putamen (Put)	(−19, 3, 9)
Left insula (lIns)	(−42, 6, 4)
Right insula (rIns)	(43, 7, 2)

**Table 3:** Group differences of default mode network functional connectivity at specific frequency bands.

Functional connectivity	0.01–0.027 Hz		0.027–0.08 Hz		0.01–0.08 Hz	
	*T*-value	*P* value	*T*-value	*P* value	*T*-value	*P* value
PCC & lLP	−2.334	0.027^*∗*^	−1.187	NS	−1.683	NS
PCC & rLP	−2.418	0.022^*∗*^	−1.696	NS	−2.186	0.037^*∗*^
liTmp & rpCBLM	−2.083	0.047^*∗*^	−1.142	NS	−2.005	NS
lLP & mdThal	−1.390	NS	−2.212	0.035^*∗*^	−2.223	0.034^*∗*^
lLP & rpCBLM	0.719	NS	−2.907	0.007^*∗∗*^	−2.495	0.019^*∗*^
rLP & rpCBLM	0.210	NS	−2.331	0.027^*∗*^	−2.357	0.026^*∗*^
liTmp & mdThal	−0.653	NS	−2.402	0.023^*∗*^	−3.015	0.005^*∗∗*^
rLP & mdThal	−1.042	NS	−2.002	NS	−2.153	0.040^*∗*^

^*∗*^ *P* < 0.05; ^*∗∗*^*P* < 0.01; NS, not significant. *T* < 0 represented the functional connectivity in *ApoEε*4 carriers was lower than the noncarriers. *T* > 0 represented the functional connectivity in *ApoEε*4 carriers was higher than the noncarriers.

PCC, posterior cingulate cortex; lLP, left lateral parietal; rLP, right lateral parietal; liTmp, left inferior temporal; rpCBLM, right posterior cerebellum; mdThal, medial thalamus.

**Table 4:** Group differences of salience network functional connectivity at specific frequency bands.

Functional connectivity	0.01–0.027 Hz		0.027–0.08 Hz		0.01–0.08 Hz	
	*T*-value	*P* value	*T*-value	*P* value	*T*-value	*P* value
lPG-ACC & rSG-ACC	2.751	0.010^*∗*^	−0.100	NS	1.079	NS
rPG-ACC & rIns	2.054	0.049^*∗*^	2.191	0.037^*∗*^	2.495	0.019^*∗*^
lIns & rIns	−2.187	0.037^*∗*^	−1.354	NS	−1.983	NS
rSG-ACC & lIns	0.698	NS	3.244	0.003^*∗∗*^	2.550	0.017^*∗*^
lPG-ACC & rIns	1.565	NS	2.117	0.043^*∗*^	2.281	0.030^*∗*^
Put & rIns	0.819	NS	2.398	0.023^*∗*^	1.670	NS

^*∗*^ *P* < 0.05; ^*∗∗*^*P* < 0.01; NS, not significant. *T* < 0 represented the functional connectivity in *ApoEε*4 carriers was lower than the noncarriers. *T* > 0 represented the functional connectivity in *ApoEε*4 carriers was higher than the noncarriers.

lPG-ACC, left anterior cingulate cortex; rSG-ACC, right ventral anterior cingulate cortex; rPG-ACC, right anterior cingulate cortex; rIns, right insula; lIns, left insula; Put, putamen.
